# Delivery of seasonal malaria chemoprevention with enhanced infection prevention and control measures during the COVID-19 pandemic in Nigeria, Burkina Faso and Chad: a cross-sectional study

**DOI:** 10.1186/s12936-022-04091-z

**Published:** 2022-03-24

**Authors:** Charlotte Ward, Abimbola Phillips, Olusola Oresanya, Gloria Olisenekwu, Ekundayo Arogunade, Azoukalné Moukénet, Honoré Beakgoubé, Vincent De Paul Allambademel, Cheick Saïd Compaoré, Adama Traoré, Jean-Bosco Ouedraogo, Yves Daniel Compaoré, Issaka Zongo, Laura Donovan, Monica Anna Decola, Helen Smith, Kevin Baker

**Affiliations:** 1 London School of Hygiene and Tropical Medicine, London, UK; 2 Malaria Consortium, Abuja, Nigeria; 3 Oxford Policy Management, Abuja, Nigeria; 4 Université Cheik Anta Diop, Dakar, Senegal; 5 Malaria Consortium, N’djamena, Chad; 6 Université De N’djaména, N’djamena, Chad; 7 Malaria Consortium, Ouagadougou, Burkina Faso; 8 Instech, Bobo Dioulasso, Burkina Faso; 9 Malaria Consortium, London, UK; 10 International Health Consulting Services Ltd, Wirral, UK

**Keywords:** Seasonal malaria chemoprevention, Malaria, COVID-19, Infection prevention and control, Children under five

## Abstract

**Background:**

Seasonal malaria chemoprevention (SMC) is a WHO-recommended intervention for children aged 3–59 months living in areas of high malaria transmission to provide protection against malaria during the rainy season. Operational guidelines were developed, based on WHO guidance, to support countries to mitigate the risk of coronavirus disease 2019 (COVID-19) transmission within communities and among community distributors when delivering SMC.

**Methods:**

A cross-sectional study to determine adherence to infection prevention and control (IPC) measures during two distribution cycles of SMC in Nigeria, Chad and Burkina Faso. Community distributors were observed receiving equipment and delivering SMC. Adherence across six domains was calculated as the proportion of indications in which the community distributor performed the correct action. Focus group discussions were conducted with community distributors to understand their perceptions of the IPC measures and barriers and facilitators to adherence.

**Results:**

Data collectors observed community distributors in Nigeria (n = 259), Burkina Faso (n = 252) and Chad (n = 266) receiving IPC equipment and delivering SMC. Adherence to IPC indications varied. In all three countries, adherence to mask use was the highest (ranging from 73.3% in Nigeria to 86.9% in Burkina Faso). Adherence to hand hygiene for at least 30 s was low (ranging from 3.6% in Nigeria to 10.3% in Burkina Faso) but increased substantially when excluding the length of time spent hand washing (ranging from 36.7% in Nigeria to 61.4% in Burkina Faso). Adherence to safe distancing in the compound ranged from 5.4% in Chad to 16.4% in Nigeria. In Burkina Faso and Chad, where disinfection wipes widely available compliance with disinfection of blister packs for SMC was low (17.4% in Burkina Faso and 16.9% in Chad). Community distributors generally found the IPC measures acceptable, however there were barriers to optimal hand hygiene practices, cultural norms made social distancing difficult to adhere to and caregivers needed assistance to administer the first dose of SMC.

**Conclusion:**

Adherence to IPC measures for SMC delivery during the COVID-19 pandemic varied across domains of IPC, but was largely insufficient, particularly for hand hygiene and safe distancing. Improvements in provision of protective equipment, early community engagement and adaptations to make IPC measures more feasible to implement could increase adherence.

**Supplementary Information:**

The online version contains supplementary material available at 10.1186/s12936-022-04091-z.

## Background

The coronavirus disease 2019 (COVID-19) pandemic represents a huge threat to the maintenance of health service delivery globally. There is potential for direct mortality from COVID-19 and indirect mortality from preventable or treatable conditions such as malaria to increase dramatically. Previous epidemics have disrupted health systems and impacted on control programmes targeting specific diseases [1]. The World Health Organization (WHO) responded with operational guidance to support countries to reorganize and safely maintain access to high-quality, essential health services in the pandemic context [2], and specific guidance for tailoring malaria interventions in the COVID-19 response [3].

This includes seasonal malaria chemoprevention (SMC), a WHO-recommended intervention for children aged 3–59 months living in areas of high malaria transmission to provide protection during the rainy season [4]. SMC, the intermittent administration of sulfadoxine–pyrimethamine (SP) and amodiaquine (AQ) during the high transmission season, has been shown to be safe, feasible, effective and cost-effective for the prevention of malaria among children under five [5, 6]. SMC is typically delivered door-to-door over a period of 4 days by trained community distributors. Many of the community distributors are community health workers, a recognized cadre of community-based primary health care worker who receive a small stipend from the government. Others are recruited specifically for the campaign, but all distributors should be from the communities they serve. The first daily dose of SPAQ is usually administered by a community distributor and the second and third daily doses of AQ are left with the caregiver to administer. In 2020, Malaria Consortium’s SMC programme targeted over 12 million eligible children in Nigeria, Burkina Faso and Chad across monthly cycles in the high transmission season [7].

Door-to-door delivery of SMC creates multiple opportunities for someone infected with severe acute respiratory syndrome coronavirus 2 (SARS-CoV-2), to transmit the virus through coughing, speaking, or exhaling, producing infective respiratory droplets that can be inhaled by those in close proximity. Infected droplets can also land on nearby surfaces or on SMC commodities such as SPAQ blister packs.

In response, operational guidance were developed to support countries to mitigate the risk against COVID-19 transmission when delivering SMC [8]. Malaria Consortium also developed a job aid [9] and a training flipbook to explain the adaptations to the campaign and guide delivery of SMC during the pandemic. The guidance also specified that caregivers should administer all SPAQ doses, with the first doses of SP and AQ administered under supervision of community distributors. Similar large-scale distribution campaigns have been implemented during previous epidemics [10, 11], but no assessment to determine adherence of infection prevention and control measures during a pandemic has been done.

Based on Donabedian’s model for assessing quality of care, availability of IPC equipment (structure), adherence to IPC measures (process) and community distributor satisfaction with IPC measures during SMC delivery (outcome) were explored [12].

## Methods

### Study design

A cross-sectional study design was used to determine adherence to IPC measures during two cycles of SMC in September and October 2020 in Nigeria, Burkina Faso and Chad. Focus group discussions were conducted with community distributors to explore their perceptions of the IPC measures and barriers and facilitators to adherence.

Here, data from each country are reported and case studies are presented to enable policy makers and SMC programme managers to observe the key challenges and successes relating to delivery of SMC during COVID-19 in areas where the SMC campaign has been implemented for at least 1 year.

### Study setting

**Fig. 1:**
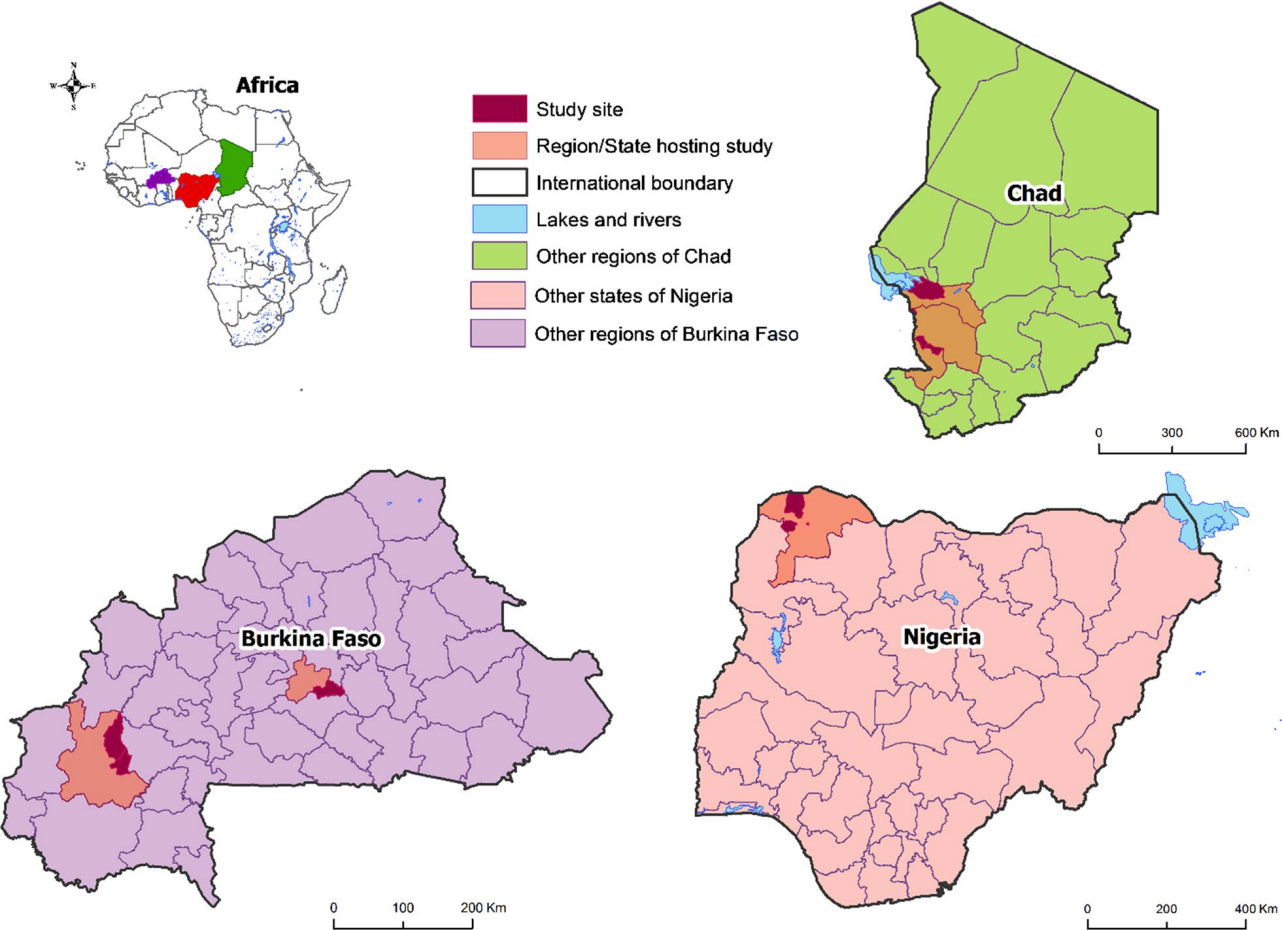
Map of the study sites and regions/states hosting the study

The study was conducted in urban centres and rural areas in Nigeria, Burkina Faso and Chad (Fig. 1). In Nigeria, the study was conducted in Sokoto state in the urban local government area (LGA) Sokoto South and rural LGAs Tangaza and Silame. SMC has been delivered in Sokoto since 2015. As of week 37 when data collection started, 159 confirmed COVID-19 cases and 17 COVID-19 related deaths had been reported in Sokoto [13]. There are 860 health facilities in Sokoto state and on average, there are five community distributors assigned to each health facility.

In Burkina Faso, the study was conducted in the urban health district of Bogodogo in the Centre region, and the rural health districts Léna and Dafra in the Hauts Bassins region. Bogodogo, an arrondissement of Ouagadougou, was added to the SMC campaign in 2019. SMC has been implemented in Dafra and Léna since 2017. The COVID-19 outbreak was concentrated around the two major cities Ouagadougou and Bobo Dioulasso. As of June 7th, 414, 28 and 0 cases of COVID-19 were confirmed in Bogodogo, Dafra and Léna, respectively. There are 72 health facilities across the three study areas and on average, there are 42.9 community distributors assigned to each health facility.

In Chad, the study was conducted in the urban health district of N’Djamena Sud in N’Djamena province, and the rural health districts of Massakory in Hadjer Lamis province and Guelendeng in Mayo Kebbi Est province. SMC has been implemented here since 2015. As of week 39 when data collection started, 1006 and 134 confirmed COVID-19 cases and 62 and 5 COVID-19 deaths had been reported in N’Djamena and Mayo-Kebbi Est provinces, respectively. There were no cases of COVID-19 reported in Hadjer Lamis province during the study period [14]. The average number of community distributors assigned to each health facility across the study areas is 33.4.

### Study sample

The study was powered to estimate the proportion of community distributors in each country who adhered to IPC measures for SMC during the COVID-19 pandemic. Using the calculation for a cross-sectional study for proportions [15], a conservative estimate of 50% adherence, desired precision of 7.5%, α = 0.05, design effect of 1.4 [16] was used, with 10% increase applied to account for loss to follow-up or missing data. A minimum sample size of n = 263 community distributors conducting SMC delivery was required for each country.

In Burkina Faso and Chad, health facilities were selected using stratified random sampling, to ensure a balanced number of urban and rural health facilities across the selected health districts. In Nigeria, multistage random sampling was used to select (a) one senatorial district per state; (b) LGAs—two rural; one urban per state; (c) health facilities.

All consenting community distributors aged 20 years or above participating in the SMC campaign at the selected health facility on the day of the observation were eligible to participate. Community distributors were selected randomly from a numbered list of all the community distributors volunteering at the health facility.

For FGDs, community distributors were purposefully sampled from health facilities across the participating regions or LGAs in urban and rural areas to form male or female only groups. Each focus group discussion comprised of community distributors from one to two purposefully selected health facilities, depending on the number of community distributors available.

### Data collection

Observations of IPC measures practiced by community distributors in the health facility and in the community were conducted. The observational tool was adapted from published tools [17, 18] that were based on WHO guidelines [19, 20]. The tool captured each opportunity or “indication” where a specific IPC action should be followed by the community distributor in accordance with the job aid [9]. For some indications, equipment was also required. The observation tool was developed on a mobile data collection platform called SurveyCTO, translated into French and Arabic where appropriate, and piloted in all countries and geographical settings prior to data collection. Data collectors were trained to spend at least three hours observing the community distributor across three time points: (i) at the health facility at the start of the day when they received equipment for IPC, (ii) in the community (visiting at least five compounds) (iii) at the health facility at the end of the day. Definitions of indications, actions and the equipment required can be found in Table 1.

**Table 1:** Definitions of infection prevention and control indications, actions and the equipment required for adherence. Source: Job Aid [9]

Step no.	Indication by domain	Action for adherence	Equipment required for adherence
Hand hygiene			
1.1	Before leaving the health facility for the community (start of day)	Wash hands for 30 s	Soap and running water or alcohol-based hand sanitizer
1.2	When entering a compound/household		
1.3	After disinfecting laminated job aid and SMC materials in the community		
1.4	After disinfecting all SMC materials and removing and disposing of face mask at health facility (end of day)		
Mask use			
2.1	Before leaving the health facility (start of day)	Put on face mask	New face mask
2.2	When entering a compound/household		
Disinfection of SPAQ~ blister packs			
3.1	At health facility (start of day)	Disinfect all SPAQ^a^ blister packs	Disinfecting wipes^b^
3.2	After 5 compounds/households (or if touched by anyone else/placed on a potentially contaminated surface)		
3.3	At health facility before storage (end of day)		
Waste management			
4.1	In the community	Dispose of used disinfecting wipes in bio-waste bag	Bio-waste plastic bag^c^
4.2	At health facility after disinfecting all SMC materials (end of day)		
4.3	At health facility (end of day)	Remove face mask and dispose in bio-waste plastic bag	Bio-waste plastic bag^c^
Safe distancing in the compound/household^d^			
5.1	During child triage	Practice safe distancing of 2 m	None
5.2	When determining child’s age		
5.3	When determining eligibility to receive SPAQ~		
5.4	During SPAQ~ administration by the caregiver		
5.5	During instructions to give day 2 and 3 AQ doses and completing record card		
5.6	When giving health promotion messages		
Ensuring community distributors are healthy			
6.1	At the health facility (start of day)	Take temperature	Infrared thermometer^e^
6.2	At the health facility (end of day)		

An indication refers to a situation where an infection prevention and control measure must be practiced to prevent the risk of SARS-CoV-2 being transmitted from one person or surface to another

^a^Sulfadoxine–pyrimethamine amodiaquine (SPAQ)

^b^Or suitable equivalent e.g. bleach and 2ply tissue paper

^c^Or suitable equivalent e.g. black polyethene bag

^d^To be marked as adherent, community distributor had to practice safe distancing at the compound for all 6 steps (5.1–5.6)

^e^Availability of infrared thermometer at health facility was not captured

Strict standard operating procedures were implemented to ensure that data collectors were as discrete as possible and did not interfere with community distributors or families, to minimize the potential for bias created by the Hawthorne effect. Data collectors were trained to use visual cues to estimate adherence to safe distancing and time taken to wash hands. To assure data quality, data collectors’ skills were evaluated during a pilot prior to data collection, data submitted in SurveyCTO were checked daily, and for a sample (5%) of observations in the community a supervisor observed the same community distributors at the same time as the data collector and inter-rater reliability was calculated.

Focus group discussions were conducted between 24 October and 20 November 2020 with community distributors who had participated in the campaign. The topic guide was designed to explore factors relating to SMC delivery in a pandemic context: (1) training; (2) challenges adhering to safe delivery of SMC; (3) equipment availability; (4) acceptability of IPC measures; (5) knowledge and awareness of COVID-19. FGDs took place in the health facilities and were conducted by trained research assistants. To facilitate open discussion, where possible male and female CDs were split into separate discussion groups. To ensure data quality, all data collection procedures and tools were piloted for one day, with separate pilots for each local language. Discussions were audio recorded.

This study was carried out by employees of Malaria Consortium and the national malaria programme in each country, together with Oxford Policy Management (Nigeria), INSTECH (Burkina Faso) and Université de N’Djaména (Chad). Authors based in Abuja, Ouagadougou and N’Djamena were involved in monitoring and evaluation activities and those in the UK were responsible for supporting implementation research.

### Data analysis

For descriptive statistics, frequencies and proportions were calculated for categorical data. Mean, standard deviation, and range were calculated for continuous data. Adherence to IPC was calculated at the indication level and then summed up to give a total adherence proportion by domain: (1) hand hygiene; (2) mask use; (3) disinfection of SPAQ blister packs; (4) waste management; (5) safe distancing in the compound/household; (6) ensure community distributors are healthy. For each indication, adherence was coded as “one” if the community distributors correctly performed the action or “zero” if they did not. If the community distributor did not have the necessary equipment to perform the action or the data collector could not see the action, it was coded as missing and excluded from the numerator and denominator.

Equipment availability analysis was done at the level of the community distributor, expressed as a proportion and described at the LGA (Nigeria) or health district (Chad and Burkina Faso) level.

Quantitative data analysis was conducted in STATA/SE version 16.

A thematic analysis was conducted for data collected during FGDs [21]. Audio recordings were transcribed verbatim and translated into English. Transcripts were read by two members of each team to generate a coding list, which was then applied to all transcripts. Teams used qualitative data analysis software: Nvivo (2018) and Dedoose version 8.0.35 (2018), or manual methods to code, manage and retrieve data. Potential themes were identified and data relevant to each theme was collated. Potential themes identified in each country dataset were then discussed together by the full team and consolidated into themes observed across all three countries. Theme names were agreed by the full team and each theme description was refined to elaborate the similarities and differences across the countries and substantiated with compelling participant quotes.

## Results

In Nigeria, forty-one public health facilities were enrolled in Sokoto state, in urban (46.3%) and rural (53.7%) areas across Sokoto South (46.3%), Tangaza (26.8%) and Silame (26.8%) LGAs. Of 259 community distributors observed, the majority (89.6%) were females with a mean age of 28.7 years (SD = 10.2). The majority (64.1%) had at least a secondary education and a mean of 3.2 (SD = 1.7) years’ experience as a community distributor for SMC (Table 2).

**Table 2:** Characteristics of community distributors enrolled in the study

Characteristics	Nigeria (N = 259)	Burkina Faso (N = 252)	Chad (N = 266^a^)
Age (years), mean [SD], min, max	28.7 (10.2), 18, 70	33.0 (9.8), 20, 63	28.8 (8.8), 20, 70
Years of experience as a community distributor, mean (SD) min, max	3.2 (1.7), 1, 7	2.8 (1.7), 0, 7	3.1 (1.8) 0, 7

	n	%	n	%	n	%
Sex						
Female	232	89.6	122	48.4	69	25.9
Male	27	10.4	130	51.6	196	73.7
Education						
No education	21	8.1	0	0.0	13	4.9
Arabic/Islamic school	31	12.0	1	0.4	17	6.4
Primary	5	1.9	27	10.7	17	6.4
Completed primary	11	4.2	37	14.7	11	4.1
Some secondary	25	9.7	125	49.6	95	35.7
Completed secondary	87	33.6	21	8.3	39	14.7
Some tertiary	26	10.0	34	13.5	54	20.3
Completed tertiary	53	20.5	7	2.8	19	7.1

^a^Descriptive data for one community distributor unavailable

In Burkina Faso, forty-six public health facilities were enrolled in Bogodogo (47.8%), Dafra (23.9%) and Lena (28.3%) health districts, in rural (56.5%) and urban (43.5%) areas. Of 252 community distributors observed, just over half (51.6%) of CDs were male, with a mean age of 33.0 years (SD = 9.8). A quarter (24.6%) of CDs had at least a secondary education and a mean of 2.8 (SD = 1.7) years’ experience as a community distributor (Table 2).

In Chad, 35 public (31.4%), private (11.4%), community (28.6%) and faith-based (28.6%) health facilities were enrolled in N’Djamena Sud (51.4%), Massakory (31.4%) and Guelendeng (17.1%) health districts, in rural (48.6%) and urban (51.4%) areas. Of 266 community distributors observed, the majority (73.7%) were male, with a mean age of 28.8 years (SD = 8.8). Under half (42.1%) of community distributors had completed secondary education or above and they had an average of 3.1 years’ (SD = 1.8) experience as a community distributor (Table 2).

Inter-rater reliability between data collectors and their supervisors was high in Nigeria (kappa: 0.77, standard error: 0.02) and Burkina Faso (kappa: 0.76, standard error: 0.03) and moderate in Chad (kappa: 0.64, standard error: 0.02).

In each country, eight focus group discussions (FGDs) were completed, across urban (n = 4) and rural (n = 4) areas with an equal number of male and female groups, with the exception of the rural areas in Burkina Faso, where four mixed male and female groups were formed.

### Observation of equipment availability and infection prevention and control adherence

#### Case study 1: Nigeria

Equipment availability was variable between LGAs and for different types of equipment, with no distinct trends between rural and urban LGAs. Overall, 67.6% community distributors received hand sanitizer and 79.9% received at least one face mask, with a much lower proportion (25.1%) receiving the recommended two new face masks. Availability of disinfecting wipes and bio-waste bags was low (Additional file 1).

Adherence to mask use was high (506 [73.3%] of 690 indications). Community distributors washed their hands on over a third of possible occasions (578 [36.6%] of 1578 indications) but rarely did so for at least 30 s (56 [3.5%] of 1578 indications). Hand sanitizer was used more frequently than soap and water. Community distributors rarely practiced exclusive safe distancing in the compound (211 [16.4%] of 1279 indications) and community distributors’ temperature was checked for 117 [22.6%] of 518 indications. Due to low availability of disinfection wipes and biowaste bags, data on compliance with disinfection of SPAQ blister packs and waste management are inconclusive (Table 3).

**Table 3:** Adherence to infection prevention and control practices, by domain

Domain	Nigeria		Burkina Faso		Chad	
	Adherence, n/N (%)	95% CI	Adherence, n/N (%)	95% CI	Adherence, n/N (%)	95% CI
Hand hygiene^a^ total	56/1578 (3.5)	2.7–4.6	165/1606 (10.3)	8.8–11.9	103/3045 (3.4)	2.8–4.1
Hand sanitizer	42/1578 (2.7)	1.9–3.6	135/1606 (8.4)	7.1–9.9	70/3045 (2.3)	1.8–2.9
Soap and water	14/1578 (0.9)	0.4–1.5	30/1606 (1.9)	1.3–2.7	33/3045 (1.1)	0.7–1.5
Any hand hygiene^b^ total	578/1578 (36.6)	34.2–39.1	994/1619 (61.4)	59.0–63.8	1362/2453 (55.5)	53.5–57.5
Hand sanitizer	528/1578 (33.5)	31.1–35.8	863/1619 (53.3)	50.8–55.8	1135/2453 (46.2)	44.3–48.3
Soap and water	50/1578 (3.2)	2.4–4.2	131/1619 (8.1)	6.8–9.5	227/2453 (9.2)	8.1–10.5
Disinfection of SPAQ blister packs total	20/78 (25.6)	16.4–36.8	51/294 (17.4)	13.2–22.2	68/402 (16.9)	13.4–20.9
Mask use total	506/690 (73.3)	69.9–76.6	1168/1344 (86.9)	85.0–88.7	1983/2437 (81.4)	79.8–82.9
Waste management total	49/98 (50.0)	39.7–60.3	102/330 (30.9)	26.0–36.2	124/302 (41.1)	35.5–46.8
Safe distancing in the compound/household^c^ total	211/1279 (16.4)	14.5–18.6	99/1249 (7.9)	6.5–9.6	135/2512 (5.4)	4.5–6.3
Ensure community distributors are healthy^d^ total	117/518 (22.6)	19.1–26.4	13/504 (2.6)	1.4–4.4	79/528 (15.0)	12.0–18.3

^a^Washed hands with soap and running water or hand sanitizer for ≥ 30 s

^b^Washed hands with soap and running water or hand sanitizer

^c^During triage AND when determining age eligibility AND SPAQ eligibility AND SPAQ administration AND instructions AND messages; in compounds where the space was not too small to be measured

^d^Take temperature with infrared thermometer at start and end of the day at the health facility

#### Case study 2: Burkina Faso

Equipment availability varied by type of equipment and by health district. The majority of the community distributors received hand sanitizer across all three health districts (96% or above). For all other types of equipment, at least 10% more community distributors in the rural district of Lena were observed receiving equipment compared to the other two districts. Notably, 59.5% of community distributors received two or more new face masks, ranging from 44.1% in Dafra to 75.9% in Lena. Disinfecting wipes were available to at least a third of community distributors in all three health districts and bio-waste bags were available to over half of community distributors (Additional file 1).

Adherence to mask use was high (1168 [86.9%] of 1344 indications). Community distributors washed their hands on over half of possible occasions (994 [61.4%] of 1619 indications) but rarely did so for at least 30 s (165 [10.3%] of 1606 indications). Hand sanitizer was used more frequently than soap and water. Among those receiving wipes and biowaste bags, there was evidence of some adherence to disinfection of SPAQ blister packs (51 [17.4%] of 294 indications) and waste management (102 [30.9%] of 330 indications). Community distributors rarely practiced exclusive safe distancing in the compound (99 [7.9%] of 1249 indications) and adherence to taking community distributors’ temperature was very low [2.6%] of 504 indications (Table 3).

#### Case study 3: Chad

Equipment availability was variable between health districts and for different types of equipment, with no distinct trends between rural and urban districts. Overall, 89.8% community distributors received hand sanitizer and 92.9% received at least one face mask, with a much lower proportion (34.2%) receiving the recommended two new face masks. Around a half of community distributors received disinfecting wipes (50.4%) and bio-waste bags (45.9%) (Additional file 1).

Adherence to mask use was high (1983 [81.4%] of 2437 indications). Community distributors washed their hands on over half of possible occasions (1362 [55.5%] of 2453 indications) but rarely did so for at least 30 s (103 [3.4%] of 3045 indications). Hand sanitizer was used more frequently than soap and water. Among those receiving wipes and biowaste bags, there was evidence of some adherence to disinfection of SPAQ blister packs (68 [16.9%] of 402 indications) and waste management (124 [41.1%] of 302 indications). Community distributors rarely practiced exclusive safe distancing in the compound (135 [5.4%] of 2512 indications) and adherence to taking temperature was quite low (79 [15.0%] of 528 indications) (Table 3).

### Findings from focus group discussions with community distributors

#### Acceptability of COVID-19 infection prevention and control measures

Community distributors in urban and rural areas of Burkina Faso, Chad and Nigeria viewed the IPC measures favourably, expressing that the equipment gave them confidence and motivation to participate in the SMC campaign despite the pandemic, typically:

“*Truly this prevention that they brought is proper and it has given us peace of mind, we know what to do because before, everyone was afraid of this sudden situation. This precaution has given us peace of mind, on top of that they added sanitizer and facemask, so we feel confident working with them… even if we get infected, we will not spread it or bring it home or to our environment*” (Nigeria_Sokoto_Urban Female_02).

“*It’s a good thing as it allows us to protect ourselves, we protect ourselves against Covid-19 and other diseases as well. And it makes us clean*.” (Burkina Faso_FGD2_F_Trame D’Accueil).

“*…at the time of the distribution, we had all the materials such as hydroalcoholic gel, gloves, and then the mask, we had all that, that’s what reassures us…*” (Chad_FDG2_M_Kamerom).

However, many community distributors were still fearful. In Burkina Faso, although community distributors generally agreed with IPC measures, many still expressed ‘a lot of fear’ of being infected with COVID-19. In Nigeria, male distributors admitted being initially fearful of becoming infected but when they were trained and assured that IPC equipment would be provided, they were put at ease. In Chad, some distributors felt compelled to use the mask for fear of being reprimanded by the police. For additional quotes see Additional file 2.

#### Impact of infection prevention and control measures on community distributors’ workload

In Burkina Faso and Nigeria, community distributors acknowledged that the biggest impact on their workload was not implementing the IPC measures, rather the time taken to explain the changed circumstances of the SMC campaign, convince reluctant caregivers about the need for additional measures and address caregiver concerns about the campaign during COVID-19.

“*The work you can finish in 30 min for example when you come and do your introduction, it will add more minutes instead of maybe 30 min, it will increase to 50 min this is because you will have to go through the measures and tell them about it step by step*” (Nigeria_Sokoto, Urban_female_02).

In Burkina Faso and Nigeria, distributors found communicating the need for IPC measures to illiterate caregivers particularly challenging. In Burkina Faso, distributors indicated that some caregivers did not fully understand the practices, especially the need for hand washing; some caregivers thought community distributors asked them to wash their hands because they were dirty.

“*Most of them are aware that it is for their own protection. But for example, when you arrive in a compound where everybody is illiterate, it's a complicated matter…We explain them, they understand, but can't really comply with the rules*.” (Burkina Faso_FGD2_F_Trame D'Accueil).

“*You arrive at someone's house and you have to wash your hands before giving medications; this can frustrate the person because it implies that their hands are not clean*.” (Burkina Faso_FGD5_F_Secteur 24).

In Chad, distributors mentioned that delivering SMC during the pandemic had led to some mistrust; some caregivers were afraid that distributors would bring COVID-19 into the household and refused compound entry on the pretext that everyone had been asked to stay at home.

“*Corona affected the distribution of CPS [SMC] […] parents are a little afraid that we are bringing this virus to them to distribute so they are afraid when approach to them for the distribution of drugs and others even outright refuse this contact. Even if we respect the distancing to give but they are afraid that corona is there so when we knock on the door many times, they respond violently, that's my opinion*” (Chad_FGD8_M_Moursal).

As a result, distributors described working additional hours in order to reach their targets for drug administration, which they said was exhausting. Most mentioned the time taken for hand hygiene for themselves and the caregiver before administering the first dose, and time to put on face masks; but they did not seem concerned by this and mostly they regarded the tasks as necessary and feasible to do.

“*You have to start raising awareness first. It is already taking time. Then you have to take the hygiene measures before doing the actual work as it was told to do. This makes it an overload of work for us*.” (Burkina Faso_FGD1_Mixed_Peele).

In contrast, community distributors in Chad felt that having to adhere to the IPC measures had a negative impact on their work and encroached on work time to the extent they felt forced ‘to do a double job’; administering SMC and raising awareness about COVID-19.

“*… the work is heavier, … it's as if we have a double mission like that, it is necessary and, to do the CPS [SMC] and it is also necessary to make the awareness of Covid so that it weighs down a little and it plays on time. Yes, it is a challenge*” (Chad_FGD7_F_Moursal).

Distributors in rural Sokoto, Nigeria explained that the state media and local town criers played a vital role in imparting information about COVID-19 prevention, creating awareness before the SMC campaign and allaying caregivers’ fears. Despite this, community distributors reported that some caregivers refused to greet them, or requested they wait outside the compound as they did not understand the rationale for the IPC measures or were worried that the visit to their home posed a threat to their safety.

#### Community distributors found face masks uncomfortable to wear

In all three countries, community distributors reported that wearing face masks throughout the working day was challenging. In Burkina Faso and Nigeria, many pointed out they had already been wearing masks for health reasons or to prevent inhalation of dust in arid regions; however, having to wear masks continuously throughout the day was their main concern. Typical discomforts community distributors experienced included:

“*There are several difficulties such as breathing problems associated with wearing masks. It happens that often you do not manage to get oxygenated air properly…*” (Burkina Faso_FGD6_Mixed_ Yegueresso).

“*yes, honestly we somehow faced challenge because…using the face mask for the fact that we were not used to it before, we put it on during work, even after until we go back to the house before they say we can remove it, we need to have it on like 6 to 7 h, we are not used to this duration*” (Sokoto, Urban_female_04).

“*Because coronavirus is there, that's how we wear the mask to distribute SMC. It squeezes and it hurts our ears. We can't breathe, even it was coronavirus that brought it all. Wearing the mask there is annoying*” (Chad_FDG1_F_Kamerom).

Some distributors mentioned that supervisors conducting spot checks facilitated consistent and continuous mask-wearing throughout the day.

Barriers to consistent mask-wearing included complaints from community members about not being able to hear them clearly when speaking. In all countries, distributors reported deliberately lowering or removing their mask when giving instructions for the drug administration but keeping the mask on to administer SMC drugs.

“*Some people demanded that it be taken off because they didn't understand what we were saying. In order for them to understand us and for the work to go well we had to take off the mask. So we had to take it off while being careful not to be seen by a supervisor. So it complicated the work a lot because we were doing it in secret, so we had to do it quickly*”. (Burkina Faso_FGD5_F_Secteur 24).

Further to this, in Nigeria, distributors mentioned that caregivers demanded to see their face when administering SMC to their children; masks were thought to be a deliberate disguise to protect distributors if adverse events occurred.

“*Well, we had challenges especially entering the houses, some parents once you knock on their door and greet them, they will start saying “you just come to give our children drugs without us knowing who you are?” So you see we will have to remove our facemask for recognition, they will even ask to know if we are the people that came the last month and we reply them, you see it is also a challenge*” (Nigeria_Sokoto, Urban_female_04).

“*Some will ask you to open your face so that they will know who they are talking to, how you look like. You can meet the owner of the house with a face mask on your face but they will insist that you should remove it so that they can know you well*” (Nigeria_Kano_Rural_male).

In Chad and Nigeria, community distributors were harassed when wearing masks—some recounted children running after them chanting, ‘the corona people’, which attracted a lot of attention and made them feel uncomfortable. In Chad, children reportedly shouted at community distributors wearing masks, as they were not familiar with face coverings.

“*We didn’t face any problem, except for the fact that whenever we enter some houses, some older men and even the younger ones that stay by road side do tag us the ‘the corona people’ and whenever they sight us from afar, they begin to say “there come the corona people”* …” (Nigeria_Sokoto, Urban_female_04).

“*The children do not know the barrier measures, when they see the community distributors, they come closer shouting ‘Mama mala’*” (Chad_FDG1_F_Kamerom).

In Burkina Faso there were reports of children running away, afraid of distributors wearing masks.

“*There is another difficulty going towards children. When you wear the mask on your way, if the children see you, they run away. They run, they don't stop. It's only your eyes that they see. They run. All of them are running. […] At a certain point in the beginning, when you arrive, the clothes on you are white and your mouth is also closed and people don't recognize you. It's when you greet in Dioula or Mooré (local language) that they say ohh*!” (Burkina Faso_FGD7_Kadomba).

#### Cultural norms made it difficult to adhere to safe distancing

In all three countries, cultural norms and traditions mean people are accustomed to greeting each other with physical contact and often spend time together in close proximity. As a result, community distributors described distancing as the most difficult measure to adhere to:

“*We were uncomfortable because we said not to greet by shaking hands with people we are already used to, as we chat and laugh together, without realizing. But if we are prevented from doing this, will we be able to be comfortable? Obviously, we won't be comfortable.*” (Burkina Faso_FGD6_Mixed_Yegueresso).

“*You know Hausa people like greetings all the time so if a man comes out from the house, the first thing he expects is for you to shake hands and if you refuse because you are trying to protect yourself, they will think you are running away from them so this is a big problem we face*…” (Nigeria_Kano_Rural_male_06).

In Nigeria, community distributors mentioned that it was difficult to observe safe distancing because they sometimes forgot, were influenced by caregivers or community members’ perceptions, and in a few instances space constraints in households precluded it.

“*Honestly, there used to be forgetfulness. There is no one that doesn’t forget things, especially when we get to a house where we will laugh with the family and the children, we do forget that we are supposed to stand afar small*” (Sokoto, Rural_female_05).

“*For example, if a caregiver notice that you are standing far from them, they will say is it because of the medicine you are doing all this? They will ask you come close, if you refuse, they will not accept the medicine*” (Kano, Rural_female_05).

In rural Sokoto state, caregivers seemed to interpret safe distancing as community distributors’ irritation or anxiety about contracting COVID-19 from them.

“*When you give a distance between caregivers, some tell us to come closer to them, some we even say are we avoiding them*” Kano_Rural_female).

Some community distributors found ways to work around the safe distancing measures. For example, in Nigeria, female distributors explained how a 1-m distance was more comfortable and feasible, so long as a face mask is worn and proper hand hygiene observed. In Burkina Faso, distributors occasionally felt obliged to shake the hands of the elderly to appear respectful, although they emphasized that they sanitized their hands immediately after. In addition, distributors in Burkina Faso were tasked with measuring nutritional status of children alongside administering SMC; using upper arm circumference in under-5s necessitated touching the child’s arm, but distributors were keen to emphasize that they disinfected the Shakir (measurement) strip before and after each use.

#### Hand hygiene adherence was sub-optimal

Community distributors recognized the importance of hand hygiene and considered hands an easy source of COVID-19 infection and transmission from touching the mouth and nose with contaminated hands. However, distributors in all three countries admitted that hand hygiene was not done as frequently or for as long as stipulated in guidelines. In Burkina Faso and Chad, distributors talked about forming a habit, although many admitted that early in the campaign they often forgot.

“*At the beginning, it was not easy at first. But in everything, the more you do it, the more you get used to it. So it was like that.*” (Burkina Faso_FGD1_Mixed_Peele).

“*Well, it's a matter of habit, these are not the measures that we are used to doing but given the arrival of this disease we knew that it is really annoying, it is really worrisome then is to enable us to protect against disease. But, most of the population does not want to apply these measures at all*” (Chad_FDG6_M_Abena).

In rural Sokoto state, Nigeria, distributors mentioned that unannounced visits by supervisors motivated them to adhere to the guidelines; some also regarded hand hygiene as a mandatory instruction and so kept to this.

“*… since it’s a promise you have taken upon yourself, you have to follow it diligently because they were appropriate and if you don’t want any problem, you just have to adhere to the directives*” (Nigeria_Sokoto_Rural_female_05).

In Nigeria, distributors explained that although they were happy to practice hand hygiene, they found it difficult to adhere to 30 s each time. A few also reported that alcohol-based sanitizers caused unpleasant skin irritations and frequent application made their hands dry. A minority expressed concern that alcohol-based sanitizer went against religious rulings that forbid use of alcohol, and they were more likely to use soap and water.

“*I can only say I did my best with the hand hygiene, but I am not certain about adhering to the 30 s rule*” (Nigeria_Sokoto_South_female_02).

“*Some Community distributor do not use the hand sanitizer. They do say it contains alcohol and so on and that because of that, their prayer is affected*” (Nigeria_Sokoto, Urban_male_01).

Availability of soap and water in households was an important challenge in all three countries. Distributors in Burkina Faso recounted having to share soap and sanitizer with households as caregivers often did not have any, and in Chad distributors found it easier to use hand gel in communities as it ‘is difficult to find soap in some homes’.

#### SMC administration by caregivers during the coronavirus pandemic

In all three countries community distributors reported having to assist caregivers to administer the first doses of SP and AQ.

“*Yes, we have to assist them during the first distribution, because they don't know how to use them. So, we demonstrate to them how they should use it*” (Nigeria_Kano_Rural_Female_).

In Burkina Faso, some caregivers recognized that their children would not accept the medicine if they had to administer it and preferred the distributor do it.

“*As they know that it's to help them that we respect the barrier measures, so some children would refuse to drink the medication if their own parents had to give them the medication. So this new method that has been adopted is a bit complicated! Unless we tease them to say we're going to give them a shot or something else, they will not take the medication with their parents!*” (Burkina Faso_FGD2_F_Trame D'Accueil).

“*In any case, many parents wanted us to administer the medication to the children because, as she said, many children didn't accept to drink the medication. But the fact of seeing the blouses, they knew it's the nurse who was and then they would easily accept!*” (Burkina Faso_FGD2_F_Trame D'Accueil).

In Burkina Faso and Nigeria, distributors also mentioned coaxing children to take the drug by singing to them or teasing them. Although some children were happy to receive the drugs from caregivers, community distributors had to step in when children cried or ran away from parents; distributors felt that this tendency was more prevalent among older children who recalled polio immunization campaigns. In Nigeria, a small number of female community distributors in urban Kano state said they carried sweets to persuade children to swallow the drugs, although this resulted in children refusing the drugs from their caregivers. In Burkina Faso, distributors mentioned that some mothers sometimes used sweets/candy to convince children to take the SMC dose.

“*Other children prefer you give them the medicine because if their mother collects it, she will deceive the child before giving him. We do lure them with candy. Before some children will collect medicine, they want you to show them candy first. I do buy candy a lot because whenever I show them, they do collect. We do tell them that if they take it, we will give them the sweet*” (Nigeria_ Kano_female_01).

“*Each woman knows her child, she tried to joke with them with candies or something else, and others intimidated the child saying: “if you don’t drink, the health worker will give you a shot” and the child would drink*.” (Burkina Faso_FGD8_Mixed_Satiri).

## Discussion

This study assessed adherence to and perceptions of IPC measures for delivery of SMC during the COVID-19 pandemic. Adherence varied across different domains of IPC, and discussions with community distributors provide insights into acceptability of the measures, barriers and facilitators to their use as well as challenges to SMC administration by caregivers. Community distributors in this study accepted the changes required to deliver SMC with IPC measures during the COVID-19 pandemic. It is encouraging that they placed value on the importance of IPC, as this has been found to increase adherence and routine adoption of measures elsewhere [22].

Observations found that adherence to hand hygiene was sub-optimal in all countries. As a primary measure to reduce the spread of COVID-19 [23] and a pre-existing recommendation for implementation of SMC, this is a concerning albeit unsurprising finding. Studies assessing health facility worker compliance with hand hygiene in Tanzania [17] and Kenya [18] found similarly low levels of compliance: 6.9% and 2.3%, respectively, although this was not in a pandemic context. Guidance for future SMC campaigns could reduce the recommended duration of hand washing to 20 s as this is within current international guidance for communities and would be more practical to adhere to, particularly when water and hand sanitizer are in short supply [24]. Access to handwashing facilities has been identified as a key factor for promoting IPC adherence [22]. For SMC, a larger volume of hand sanitizer could also reduce the discomfort experienced by community distributors using insufficient amounts of hand sanitizer to wash their hands and encourage them to wash for longer, in a context where soap and running water is not always available [25].

The majority of community distributors were observed wearing their face mask. Higher availability of face masks and government-imposed obligations to wear them are factors facilitating adherence to mask wearing. Concerns about wearing face masks including discomfort, reduced ability to communicate and reports of harassment were shared during FGDs. Their discomfort and the fear they can illicit, particularly in children, has been reported elsewhere [22]. A recent study found that introduction of face shields significantly reduced COVID-19 transmission to community health workers [26] and could offer a more comfortable alternative in a setting where face masks are often negatively perceived and disrupt community distributors’ ability to communicate with families.

Observations suggest that practicing 2-m safe distancing exclusively in the compound was challenging in all three countries. A recent paper discusses how cultural values including solidarity between extended family groups makes respecting social distancing during COVID-19 in west African societies particularly challenging [27]. Community distributors for SMC are often members of the community and are known to the families, making it difficult to respect both safe distancing and cultural values such as physical greetings. A recent review suggests that maintaining at least 1-m safe distancing offers protection from infection, which, in space-constrained areas could be more practical and culturally acceptable [28].

In the context of the pandemic, normal community engagement activities such as sensitization meetings with local leaders and community members and dissemination of information through town announcers and media were cancelled or adapted [29]. Qualitative data suggest that there was a lack of awareness in the community about the importance of IPC measures and this greatly impacted on the workload of community distributors, particularly when caregivers were illiterate. Future community engagement approaches should start as early as possible and consider visual and audible information such as posters and radio jingles to support illiterate caregivers to understand the measures. Campaign planners could also reduce the target number of children allocated to each community distributor to reduce the additional workload experienced by community distributors during the campaign which is essential for their wellbeing, motivation and retention and has been reported as a barrier to adherence elsewhere [22].

The international guidance and standards for safe implementation of SMC rapidly developed for the 2020 campaign were based on the best available evidence at the time. It is evident that there were differences between these and national IPC standards. For example, in Burkina Faso, community distributors were also responsible for anthropometric measurements to assess nutritional status which compromised efforts to ensure safe distancing [personal communication, SMC research coordinator]. In Chad, the health authorities were recommending safe distancing of only 1-m outside the SMC campaign [personal communication, SMC data analyst]. Such discrepancies between national and international guidance may have reduced adherence as has been reported elsewhere [22].

The observational tool used in this study was based on direct observations which could have led to community distributors altering their behaviour because they are being observed, known as the Hawthorne effect. The observation tool was also restricted to the SMC IPC activities stipulated in the job aid and based on international guidance. Due to the rapidly changing nature of the COVID-19 pandemic, there were local adaptations to the types of equipment used and the order with which some of the SMC activities took place that could not be captured in the observation tool. Efforts were made to accommodate local adaptations where possible in the pilots, but it is plausible that the indicators reported here underestimate the actual compliance where equivalent equipment were used or IPC events took place at a different time to when they were observed. Mask use should have been practiced continuously, but was measured at discrete time points and could be an overestimation of the actual compliance over time. Due to low availability of biowaste bags and disinfecting wipes in Sokoto State, Nigeria, there was a high proportion of missing data for these domains, making the adherence results inconclusive. Finally, the tool did not capture availability of the infrared thermometer at the health facility or soap and water at the health facility or compound, and results should be interpreted with this caveat in mind.

It is worth noting that the quantitative findings presented here relate to IPC adherence and equipment availability in the latter two cycles of SMC. These could have changed within the 4-month campaign, influenced by a range of factors including time since training, community distributors’ awareness and perceptions of COVID-19 and changes to the availability of equipment. Qualitative findings allow the reader to gain insight into additional issues occurring throughout the 4-month campaign.

## Conclusion

Community distributors in this study accepted the changes required to deliver SMC with IPC measures during the COVID-19 pandemic. Adherence varied across domains of IPC, but was largely insufficient, particularly for hand hygiene and safe distancing and key barriers to adherence were identified.

Future campaigns could consider reducing hand washing time, reducing the safe distancing recommendation to one meter and implementing the use of face shields to make the measures more feasible to adhere to. Early and continuous community engagement and further training on how to communicate the importance of the IPC measures could also help relieve the dual burden placed on community distributors to sensitise the communities to the SMC campaign and raise awareness about COVID-19 prevention.

## Supplementary Information

**Additional file 1: Table S1.** Number and proportion of community distributors observed receiving equipment, by country and local government area (Nigeria) or health district (Burkina Faso and Chad).

**Additional file 2.** Additional quotes.

## Data Availability

The data that support the findings of this study are available from the corresponding author upon reasonable request.
