# Supplementary material for: Delivery of seasonal malaria chemoprevention with enhanced infection prevention and control measures during the COVID-19 pandemic in Nigeria, Burkina Faso and Chad: a cross-sectional study
Source: Malar J. 2022 Mar 24;21:103. doi: 10.1186/s12936-022-04091-z (PMC8943494; doi:10.1186/s12936-022-04091-z)
Supplement: Supplementary file 1 — Additional file 1: Table S1. Number and proportion of community distributors observed receiving equipment, by country and local government area (Nigeria) or health district (Burkina Faso and Chad). [file 12936_2022_4091_MOESM1_ESM.docx]

# Additional file 1

Table S1 Number and proportion of community distributors observed receiving equipment, by country and local government area (Nigeria) or health district (Burkina Faso and Chad)

| **Equipment** | **Response** | **Nigeria** | | | | | | | | **Burkina Faso** | | | | | | | | **Chad** | | | | | | | |
| --- | --- | --- | --- | --- | --- | --- | --- | --- | --- | --- | --- | --- | --- | --- | --- | --- | --- | --- | --- | --- | --- | --- | --- | --- | --- |
|  |  | **Sokoto South (N=138)** | | **Tangaza (N=61)** | | **Silame (N=60)** | | **TOTAL** | | **Bogodogo (N=130)** | | **Dafra (N=68)** | | **Lena (N=54)** | | **TOTAL** | | **N'Djamena (N=135)** | | **Hadjer Lamis (N=84)** | | **Mayo Kebbi Est (N=47)** | | **TOTAL** | |
|  |  | **Value** | **%** | **Value** | **%** | **Value** | **%** | **Value​** | **%** | **Value​** | **%** | **Value​** | **%** | **Value​** | **%** | **Value​** | **%** | **Value​** | **%** | **Value​** | **%** | **Value​** | **%** | **Value​** | **%** |
| **Hand sanitiser received** | Yes | 101 | 73.2 | 46 | 75.4 | 28 | 46.7 | 175 | 67.6 | 129 | 99.2 | 68 | 100.0 | 52 | 96.3 | 249 | 98.8 | 122 | 90.4 | 70 | 83.3 | 47 | 100.0 | 239 | 89.8 |
| **No. face masks received** | None | 15 | 10.9 | 3 | 4.9 | 13 | 21.7 | 31 | 12.0 | 1 | 0.8 | 0 | 0.0 | 1 | 1.9 | 2 | 0.8 | 7 | 5.2 | 5 | 6.0 | 5 | 10.6 | 17 | 6.4 |
|  | 1 | 77 | 55.8 | 38 | 62.3 | 27 | 45.0 | 142 | 54.8 | 40 | 30.8 | 34 | 50.0 | 4 | 7.4 | 78 | 31.0 | 90 | 66.7 | 49 | 58.3 | 14 | 29.8 | 153 | 57.5 |
|  | 2+ | 42 | 30.4 | 9 | 14.8 | 14 | 23.3 | 65 | 25.1 | 89 | 68.5 | 34 | 50.0 | 49 | 90.7 | 172 | 68.3 | 37 | 27.4 | 30 | 35.7 | 27 | 57.4 | 94 | 35.3 |
| **Condition of face mask** | New | 114 | 82.6 | 45 | 73.8 | 38 | 63.3 | 197 | 76.1 | 119 | 91.5 | 62 | 91.2 | 45 | 83.3 | 226 | 89.7 | 119 | 88.1 | 74 | 88.1 | 41 | 89.1 | 234 | 88.0 |
| **At least 2 new face masks received** | Yes | 42 | 30.4 | 9 | 14.8 | 14 | 23.3 | 65 | 25.1 | 79 | 60.8 | 30 | 44.1 | 41 | 75.9 | 150 | 59.5 | 34 | 25.2 | 30 | 35.7 | 27 | 58.7 | 91 | 34.2 |
| **Disinfecting wipes* received** | Yes | 19 | 13.8 | 6 | 9.8 | 1 | 1.7 | 26 | 10.0 | 44 | 33.9 | 23 | 33.8 | 31 | 57.4 | 98 | 38.9 | 55 | 40.7 | 45 | 53.6 | 34 | 73.9 | 134 | 50.4 |
| **Bio-waste bag^ received** | Yes | 32 | 23.2 | 31 | 50.8 | 7 | 11.7 | 70 | 27.0 | 76 | 58.5 | 43 | 63.2 | 45 | 83.3 | 164 | 65.1 | 57 | 42.2 | 40 | 47.6 | 25 | 53.2 | 122 | 45.9 |
| *Or equivalent – bleach and 2ply tissue paper | | | | | | | | | | | | | | | | | | | | | | | | | |
| ^Or equivalent – black polyethene bag | | | | | | | | | | | | | | | | | | | | | | | | | |
