# Supplementary material for: Delivery of seasonal malaria chemoprevention with enhanced infection prevention and control measures during the COVID-19 pandemic in Nigeria, Burkina Faso and Chad: a cross-sectional study
Source: Malar J. 2022 Mar 24;21:103. doi: 10.1186/s12936-022-04091-z (PMC8943494; doi:10.1186/s12936-022-04091-z)
Supplement: Supplementary file 2 — Additional file 2. Additional quotes. [file 12936_2022_4091_MOESM2_ESM.docx]

# additional file 2

## Additional quotes

Box 1.1 Acceptability of COVID-19 infection prevention and control measures

| **Burkina Faso**  “I can see that there were challenges, frankly; that's why we had to keep ourselves clean first! Because in the past we touch the children randomly. Frankly, I think it made us clean! And we avoided dust!” *(FGD3_F_Trame D'Accueil)*  N2: "As it is in the context of health, then let it protect us. What protects us against is an obligation that we implement "(*FGD1_Mixed_Kadomba)*  “Yes, we trust these measures because they were announced on television; we made people aware of the need to wash their hands and wear masks, and we saw that it was the same thing we were doing.” (FGD2_F_Trame D'Accueil)  “It's like I said, it's good! It's to protect us against diseases, so it's good! It's tiring but it's good! Yes!” (FGD2_F_Trame D'Accueil)  “In any case, it's the same thing. I come back to what she said. It's a good thing as it allows us to protect ourselves, we protect ourselves against Covid-19 and other diseases as well. And it makes us clean.” (FGD2_F_Trame D'Accueil)  Yes it's really complicated because there are people when you come into their concession they are ready to shake your hand so it's up to you to refuse and tell him that it is forbidden to greet each other while hugging hands. You take the opportunity to start raising awareness. This is where we are moving. There are people when they cross you the sign so it's really hard for us? (FGD6_Mixed_Yegueresso)  “This is good because before we “struggled” with mothers to administer drugs, we could come into contact with other infectious diseases; is not it? but with the coronavirus with all these new measures, and we weren't touching the children and we weren't getting close to them; also that mothers know what to do about hygiene with children. This prevents us from being contaminated by diseases” (FGD1_Mixed_Peele)  **Nigeria**  “Truly this prevention that they brought is proper and it has given us peace of mind, we know what to do because before, everyone was afraid of this sudden situation. This precaution has given us peace of mind, on top of that they added sanitizer and facemask, so we feel confident working with them. We believe that by God's grace we will be protected, even if we get infected, we will not spread it or bring it home or to our environment” (Sokoto_ Urban Female _02).  “These are very important measures I tell you. It is very necessary for keeping us safe, I think it should not just be for COVID alone even, because we used to enter a lot of places and talk to different persons, we should have been doing this before now” (Sokoto_ Urban_female_06).  “The imposed rule is of benefit. These measures of corona virus have been very useful, everybody was happy about it, not even only us working that is happy about it, even some people not working with us when they see us doing it, they normally watch and they are also learning it and are really happy about it” (Sokoto_Rural_female_07)  “What amazes me about this year's work is the clothing to identify us as Community distributor. I never appreciated that I was a health worker or a drug administrator for prevention of sickness until this year. Because last year there was no means of identification but this year there is face mask, your clothes then there is sanitizer. Anyone that sees you with that thing sees you with new eyes and knows who you are immediately. Even small children know that we are health workers” (Sokoto, Urban_male_01).  **Chad**  “Which reassures us, during this ...at the time of the distribution, we had all the materials such as hydroalcoholic gel, gloves, and then the mask, we had all that, that's what reassures us that we are in the process of do, hmm” (FDG2_M_ Kamerom)  “If I am attacked by the corana virus, another person could be infected too. And when I wear the mask, I am protecting myself and I am protecting others as well. And when I wear this I feel comfortable, nothing bothers me” (FDG2_M_ Kamerom)  “I wear because this disease exists. To save your life there, you have to wear this, you have to apply all these measures to avoid this disease” (FGD7_F_Moursal)  “It’s not difficult for us. We are doing our best and that is also to protect ourselves against this disease. It is mandatory. One should not touch the nose, mouth etc. So, we wear our masks normally and we greet each other with our mouths. And then we apply the gel and whoever writes on the doors there also uses the gel once they have written. We're used to that!” (FGD7_F_Moursa)  "We know that there are protective measures. So being the nursing staff we are not afraid so we go out to help people by giving medicines to children. Even if there is COVID19, we are not already protected " (FGD4_M_ Mogrom)  “Now with the corona virus, we often wash our hands and wear masks that protect us from a lot of other illnesses. These measures are very important, I assure you” (FDG2_M_Kamerom)  “Even here at the health center, these measures have helped us in our work. Thanks to corona virus, we wear masks, we use hydroalcoholic gels and we wash our hands. We also put hand washing facilities at the entrance to the health center for people to use. In addition, we tell them not to group together at the risk of being contaminated or spreading the corona virus disease. That is also a good one” (FDG1_F_Kamerom) |
| --- |

Box 2.1. Impact of infection prevention and control measures on community distributor’s workload

| **Burkina Faso**  “How coronavirus affected work, this is called, overwork. Because you have to start raising awareness first. It is already taking time. Then you have to take the hygiene measures before doing the actual work as it was told to do. This makes it an overload of work for us." (FGD1_Mixed_Peele)  “Yes, there has been an increase in our tasks. If we leave, first of all we have to raise awareness about malaria by giving them advice such as: sleeping under mosquito nets, cleaning the premises, protecting themselves against mosquitoes. In addition, we must also raise awareness about COVID by telling them to wash their hands with soap after returning from the market, to wear a nose mask when going to town, and to keep a distance of 10m.... of 1m. Before there was no such thing, which was an increase in the number of tasks, so it becomes two tasks.... Rather three tasks, since it is necessary to add an awareness raising on malnutrition.” (FGD5_F_Sector 24)  “What I would like to list as the difficulties is that before the coronavirus disease, we were doing awareness raising on SMC for money. We still continue to do not only the same work but also to carry out activities related to the coronavirus disease, however the amount received has not changed. The volume and the workloads have increased except for the money and that is what we cannot understand.” (FGD6_Mixed_ Yegueresso)  “But because of these additional measures the work of the campaign has become tiring, but we have made a commitment to help our people, so we cannot betray them, that's why we do the work, otherwise it's very tiring.” (FGD8_Mixed_Satiri)  “Most of them are aware that it is for their own protection. But for example, when you arrive in a compound where everybody is illiterate, it's a complicated matter. With Illiterate people, it's a bit complicated! We explain them, they understand, but can't really comply with the rules.” (FGD2_F_ Trame D'Accueil)  “You arrive at someone's house and you have to wash your hands before giving medications; this can frustrate the person because it implies that their hands are not clean.” (FGD5_F_Secteur 24)  **Nigeria**  “The work you can finish in 30minutes for example when you come and do your introduction, it will add more minutes instead of maybe 30minutes, it will increase to 50minutes this is because you will have to go through the measures and tell them about it step by step” (Sokoto, Urban_female_02).  “honestly, my work is taking much time but it has not become a problem for me because what was added is important for the prevention of the virus, the additional work is for prevention not for any other thing and it didn’t cause us anything” (Sokoto, Urban_female_04).  “It is not a problem for me honestly. The only thing that became an issue to me is the additional time of closing we were closing by either 12:30 or 12pm but now we have additional 35-40 minutes to it, and you see that’s not an issue. Honestly, we didn’t take it as an issue” (Sokoto, Urban_female_04).  “You will see a particular work that you are supposed to round up in 5minutes you will end up rounding up in 10minutes, seriously houses that you are expected to cover per day, because of all those issues you will be unable to complete it” (Sokoto_Rural_Female)  **Chad**  “These measures take a long time for work. They delay labour because at every moment you have to apply the hydroalcoholic gel, wash your hands, do this and that. So it doesn’t work out. In a household where you find 5 to 6 children instead of 3 to 5 minutes, you will do 15 to 20 minutes. All this is in relation to the application of these measures” (FGD7_F_Moursal)  "... the work is heavier, that's what I was saying, it's that workload, because before, we were supposed to simply administer only the drugs, but here now it's as if we have a double mission like that, it is necessary and, to do the CPS [SMC] and it is also necessary to make the awareness of covid so that it weighs down a little and it plays on time. Yes, it is a challenge” (FGD7_F_Moursal)  “It’s a bit very, very difficult, for before I didn’t work in CPS [SMC] but I saw that it’s easy but the year 2020 when covid has happened is very very difficult. Give the child a tablet if the father or mother is first of all going to wash his own hand with gel, he will put on the glove and the glass or his cup to give the child. Two, to carry the tablets in the bag to go to the field to serve, it is difficult. Those in the center go to you at 3 p.m. and work for example a single household you will find children and to serve it is difficult. And make the report, when you go to make the report 4 pm” (FDG4_M_Mogrom)  “The barrier measures disrupted our distribution work a lot, but we are already used to these measures and we cannot also say no or give up. As long as the disease is still there we will always apply only. If the disease is gone, we will also leave some things. But we see the easy ones like applying gel there we can” (FDG4_M_Mogrom)  "What weighs on us here is that before we have only one CPS [SMC] activity and the 2,500 XAF [remuneration] there itself. There is no problem. Whereas now it is double activity. You go to suffer until and you come back to the 2,500 XAF again, that's what is difficult for us. We make it difficult but above all it is for our health, we just want to stop the disease. We work on two lines but we pay on one line, that's what is not good” (FDG4_M_Mogrom)  “It bothered us a lot on the job. It bothered us a lot on the job this year. Wash hands with soap, wear a mask, no greeting in hand, distance. Before entering a compound, you have to distance yourself and the head of the household. It bothered us a lot about covid-19” (FDG4_M_Mogrom)  "Yes I think that with the arrival of covid-19, we have a lot of difficulties. When we arrive in a household, we disinfect everything before starting, I think it takes us a lot of time but we have to do it because covid is there, so we have a lot, it took us a lot of time” (FDG6_M_Abena)  Corona affected the distribution of CPS a bit in that it caused a bit of mistrust […] parents are a little afraid that we are bringing this virus to them to distribute so they are afraid when we are approach to them for the distribution of drugs and others even outright refuse this contact. Even if we respect the distancing to give but they are afraid that corona is there so when we knock on the door many times, they respond violently, stuff like that, that's my opinion” (FGD8_M_Moursal) |
| --- |

Box 2.2. Community distributors found facemasks uncomfortable to wear

| **Burkina Faso**  “There are several difficulties such as breathing problems associated with wearing masks. It happens that often you do not manage to get oxygenated air properly. But, with all these difficulties we were able to do our job thanks to God.” (FGD6_Mixed_ Yegueresso)  “At the beginning it wasn't easy because breathing under the nose mask, when you're not used to it, was a problem. So, when we were on the street, because there's some...space between the compounds, when we went out of a compound, we put the mask down like this, when we got to a compound, to talk to the parents too, we would get them up.” (FGD3_F_Trame D'Accueil)  “There is another difficulty going towards children. When you wear the mask on your way, if the children see you, they run away. They run, they don't stop. It's only your eyes that they see. They run. All of them are running. […] At a certain point in the beginning, when you arrive, the clothes on you are white and your mouth is also closed and people don't recognize you. It's when you greet in Dioula or Mooré (local language) that they say ohh!” (FGD7_Mixed_Kadomba)  “In my opinion, we cannot respect all the measures. For example, wearing masks, not everyone can wear the mask for hours, like me, if I wear the mask 1 hour later I have to take it off.” (FGD8_Mixed_Satiri)  “In the beginning, when you put on the mask, it was difficult to breathe! We couldn't breathe well, but now that we're used to it, we can breathe without any problem.” (FGD2_F_Trame D’Accueil)  “Some people demanded that it be taken off because they didn't understand what we were saying. In order for them to understand us and for the work to go well we had to take off the mask. So we had to take it off while being careful not to be seen by a supervisor. So it complicated the work a lot because we were doing it in secret, so we had to do it quickly”. (FGD5_F_Secteur 24)  **Nigeria**  “I do forget to use it at times, and it disturbs me. When they gave it to me after we started work it do disturb me so I removed it, when I removed it and went to work till, I come back honestly, I would forget putting it on. There was even a time my colleague asked me where my face mask is I said is in my pocket they now told me I must be putting it on. To me I felt it was disturbing me” (Sokoto, Urban_male_01).  “honestly my face mask has finished before I finish work, sometimes my child is crying and so he drag the face mask and tear it, that’s the only reason for which I do not wear it” (Sokoto, Urban_female_02).  “Well when we were given facemask at first, I was honestly feeling peppery on my face when I started using it and my breath will be doing as if it will cease but I endured because they said the facemask protects us and I get used to it that way” (Sokoto, Urban_female_03).  “We didn’t face any problem, except for the fact that whenever we enter some houses, some older men and even the younger ones that stay by road side do tag us the ‘the corona people’ and whenever they sight us from afar, they begin to say “there come the corona people” some tell us that the boxes we carry and the facemask we wear resemble cows that are tied to ropes and cow isn’t human being” (Sokoto, Urban_female_04).  yes, honestly we somehow faced challenge because, we had some challenges using the face mask for the fact that we were not used to it before, even though we were using it in the past but now we use it often, we put it on during work, even after until we go back to the house before they say we can remove it, we need to have it on like 6 to 7 hours, we are not used to this duration” (Sokoto, Urban_female_04).  “Well, we had challenges especially entering the houses, some parents once you knock on their door and greet them, they will start saying “you just come to give our children drugs without we knowing who you are? so you see we will have to remove our facemask for recognition, they will even ask to know if we are the people that came the last month and we reply them, you see it is also a challenge” (Sokoto, Urban_female_04).  **Chad**  "At first it is not easy to bear; I wear it as if the breathing will cut like that. So soon I shoot, if I want to drink I have to shoot up after now I see it's good already we are already used to it” (FDG5_F_Abena)  “Some, we leave from the house with. So it's already (whispers) from the house as soon as you leave at 6am sometime before you get there. It wasn’t easy, why, because we’re not used to it a few times it’s like it suffocates us, it’s a reflex by the way. We used to want to shoot like that first ... and when we see the supervisors behind us first we try to pull that out. It wasn't easy because the time is long and to stay like that with there, suddenly it suffocates us so it was not easy for me” (FDG5_F_Abena)  "My nose hurts [wearing a mask] (Laughs ...)" (FDG1_F_Kamerom)  “Because coronavirus is there, that's how we wear the mask to distribute SMC. It squeezes and it hurts our ears. We can't breathe, even it was coronavirus that brought it all. Wearing the mask there is annoying” (FDG1_F_Kamerom) |
| --- |

Box 2.3. Cultural norms made it difficult to adhere to social distancing

| **Burkina Faso**  “We were uncomfortable because we said not to greet by shaking hands with people we are already used to, as we chat and laugh together, without realizing. But if we are prevented from doing this, will we be able to be comfortable? Obviously, we won't be comfortable. ((Laughter)) This is the aspect that is complicated”. (FGD6_Mixed_Yegueresso)  “There were elderly people in some compound and when we entered their compounds, we had to shake their hands. But as there is a question of distance, we really had to take enough time to explain to them why we should no longer shake hands. Otherwise others would say you disrespected them.” (FGD8_Mixed__Satiri)  “Because there was work pressure, which increased slightly with the increase in workloads due to the measures that were there. We were familiar with the people. That was no longer possible. A person we've known since, when we walk into his yard, the child runs towards us and we could even take him. But now, when the child runs towards us, we look for each other, because we do everything to not be seen by our supervisor. There is all that and I was frankly not comfortable” (FGD4_Mixed_Sector 24)  “We cannot be comfortable. Especially with social distancing, when you walk into a yard and explain to the person that you need a distance of one meter between him and us. The face of the person suggests that they are being neglected, when it is not. We have to respect that, because that is what we have been told. ”(FGD4_Mixed_Sector 24)  “I can say that my predecessor has already said. If between us human we cannot approach each other, it is a big difficulty first. You go into someone's house to heal and you don't want to approach the person, this is a big difficulty (laughs) while wanting you to understand each other. Can you understand each other? Even if the person accepts, they have accepted, if not really…, it is a great difficulty”. (FGD6_Mixed_Yegueresso)  **Nigeria**  “Honestly, there used to be forgetfulness. There is no one that doesn’t forget things, especially when we get to a house where we will laugh with the family and the children, we do forget that we are supposed to stand afar small” (Sokoto, Rural_female_05)  “Like that of social distancing, since you know the person and they know us, it is hard not to come, sit and discuss and even shake” (Sokoto_Rural_male_)  **Chad**  "Where we have difficulties there is social distancing because little children they don't know; they don't respect the barrier rules. This is where we had the difficulty with social distancing there alone” (FGD2_M_Kamerom)  “Especially with the coronavirus measures it is very difficult with Africans. With our greeting there so that we do not greet each other with our hands, it is not easy” (FDG1_F_Kamerom)  “What lasts with us is that before when we come to a household we sit on the mat with all the parents, we get comfortable giving SMC and we continue in another household. Now the parents look at us like we are wizards who come to their homes. They are suspicious of us, they stay away from us, they are afraid of us and we too do the same. With corona there someone is always alone like an evil spirit that is in a house. So, it’s really hard. We want to make coronavirus go away definitively” (FDG1 _F_ Kamerom)  “Now when we go down to the field we stay away from our sisters. Even hand-to-hand greetings there, we don't. Before we took the children and we asked the questions how are you my dear. You have to know how to talk to the child before giving it. Sometimes when you get to the door even there, when the children see you they run to go inside. This is due to COVID19. They are afraid of the disease being brought to them” (FGD8_M_Moursal)  "It is not easy as she says there true because as a good Chadian there you have to shake hands, because when you only greet like that, the person tells you that she is not in the car. So you have to shake hands and given the illness, in doing this some people find that you may be neglecting them, so a kind, that's it!” (FDG5_F_Abena)  “The embraces. The greeting…the hand-to-hand greeting is what she just said” (FDG5_F_Abena)  "... without saying hello, shaking hands is a bit difficult. Because when you find your sister, you have to shake hands first. And when you don't shake your sister's hand, it bothers you a lot and we are used to (laughs) on both sides it bothers). And since we're already used to his measurements now, I think it's good” (FDG5_F_Abena)  "It's not easy, we try to distance ourselves but mothers, they will always come and want to see what we are doing, sometimes they approach very close to us and it's not easy, that been a challenge” (FDG5_F_Abena) |
| --- |

Box 2.4. Hand hygiene adherence was sub-optimal

| **Burkina Faso**  “At the beginning, it was not easy at first. But in everything, the more you do it, the more you get used to it. So it was like that.” *(FGD1_Mixed_Peele)*  “That's what I meant he said. This is true between two or three concessions, it can happen that the work (respecting the measures), we forget a little, a little; is not it? But gradually, once in the concession, very quickly, we disinfect with the gel, we protect ourselves well before starting. We greet, we give the purpose of our visit. So, after two or three days, it becomes routine; therefore different from the first days. Handwashing on the first day ... Because it's a new habit, because we don't wash our hands so much except to eat. So for a start, we are tempted to say that the work is difficult." *(FGD1_Mixed_Peele)*  “I think it's a reflex, at the beginning it's a bit difficult but the fact that it's a reflex means that you get used to it.” *(FGD2_F_Trame D'Accueil)*  **Nigeria**  “Yes ma since it’s a promise you have taken upon yourself, you have to follow it diligently because they were appropriate and if you don’t want any problem, you just have to adhere to the directives” (Sokoto_Rural_female_05)  "If you come out from 1, 2 houses you will have to wash your hands again, and if you write you still have to wash your hands again. So, we are already used to washing of hands but not as many times as they said. Seriously, we all know it's a protection, and we know washing hands won’t harm us, it’s a protection against Corona virus, but it is too often” (Sokoto_ Rural_male)  “Some Community distributor do not use the hand sanitizer. They do say it contains alcohol and so on and that because of that, their prayer is affected” (Sokoto, Urban_male_01)  “I can only say I did my best with the hand hygiene, but I am not certain about adhering to the 30 seconds rule” (Sokoto_South_female_02).  “Well, by the grace of God, though I cannot say I maintained the timing all through, but I tried my best in estimating it since it is only God that is perfect” (Sokoto_urban_female_04).  **Chad**  “Well, it's a matter of habit eh, these are not the measures that we are used to doing, but given the arrival of this disease we knew that it is really annoying, it is really worrisome then is to enable us to protect against disease. But most, most of the population does not want to apply these measures at all” (FDG6_M_Abena)  "Hand washing is a bit complicated, but using hydro alcoholic gel is easier because you just need to press and it doesn't take long. Now, hand washing sometimes makes it difficult to find soap in some homes. Some people cannot get soap at home” (FGD7_F_Moursal)  "Already there is no shortage of water in N’Djamena, if only it was to the north where they may be lacking water, but in N’Djamena, water is easily found. So if it's washing your hands for 30 seconds, that's possible anyway. Yes I wash my hands but I didn’t stop the clock but I tell myself that it is possible, washing my hands for 30 seconds is possible” (FDG5_F_Abena)  "If there is no hydroalcoholic gel we are looking for soap and if there is no soap then we have the gloves we are wearing [...]” (FDG2 _M_Kamerom)  “…Sometimes, when we forget to wash our hands, children or parents of children ask us to wash our hands before entering their compound” (FDG1_F_ Kamerom) |
| --- |

Box 3.1. SMC administration by caregivers during the coronavirus pandemic

| **Burkina Faso**  “As they know that it's to help them that we respect the barrier measures, so some children would refuse to drink the medication if their own parents had to give them the medication. So this new method that has been adopted is a bit complicated! Unless we tease them to say we're going to give them a shot or something else, they will not take the medication with their parents!” *(FGD2_F_Trame D'Accueil)*  “In any case, many parents wanted us to administer the medication to the children because, as she said, many children didn't accept to drink the medication. But the fact of seeing the blouses, they knew it's the nurse who was and then they would easily accept!” *(FGD2_F_Trame D'Accueil)*  “Each woman knows her child, she tried to joke with them with candies or something else, and others intimidated the child saying: "if you don't drink, the health worker will give you a shot" and the child would drink.” *(FGD8_Mixed_Satiri)*  “Some would tell that their children had some reactions the previous years after taking the drugs, and then they are not ready to let them receive the medication any more. But we tried to make them understand that it's not how they should consider things!” *(FGD2_F_Trame D'Accueil)*  “There are others, when we arrived in the compound, we knew there were children, but they would tell you, there were no children! But we knew there were children! But they would tell you... It's a case of refusal!” *(FGD3_F_Trame D'Accueil)*  “Just recently in my area I discovered another situation in 3 compounds. The mother said that the child is permanently under treatment, it was the neighbours who denounced it. They have all sorts of pretexts not to take for their children.” *(FGD5_F_Secteur 24)*  **Nigeria**  “Alright, there are those that will prefer taking from their parent because they do not know us and sometimes, the parents have to struggle with the child before he will agree to take it” (Sokoto, Urban_ female_03).  “After they will consent to our activities, we have to drop our bags use water to wash our hands and then also give the mother of the child to do same, after which she will then have to get us a container to soak the drugs, she will give her child, we will take about 30minutes if the child didn’t cry before we are done” (Sokoto, Urban_female_04).  **Chad**  “Corona affected the distribution of CPS a bit in that it caused a bit of mistrust […] parents are a little afraid that we are bringing this virus to them to distribute so they are afraid when we are approach to them for the distribution of drugs and others even outright refuse this contact. Even if we respect the distancing to give but they are afraid that corona is there so when we knock on the door many times, they respond violently, stuff like that, that's my opinion” (FGD8_M_Moursal)  “Speaking of distancing, that is standing a meter away. But compared to the distance of 1m there it is not possible because you are the one who serves. And while serving, you cannot stay 1m away. the child comes close to you before you give him his medicine. So your distance there, I don't think there is” (FDG4_M_Mogrom)  "There it is not easy, that is to say, she does not follow exactly what she is asked to do. We might ask to bring, how shall I put it, take the medicine and, take two pills out for the first dose, sometimes she doesn't do exactly what she's asked to do -there, maybe she even removes three. Effectively we keep saying that no, no it should have been two and not three so we remove the plate and give it a plate and give it another plate so that she takes and respects that. "(FGD8_M_Moursal)  "Well, in my opinion in the field, there are some parents who also do not understand, instead of doing the other, administering the child in your presence, they say give me the product and then I will administer it. after and we do not know what she will do after. "(FGD8_M_Moursal)  I am in accepting caregivers. When we arrive in some households, some even refuse to welcome us and others we are there we say: cok! cok! cok! cok! cok! cok they refuse to go out we don't know is it because of this disease that they are afraid or I don't know they refuse these drugs, we don't know. So, on this point we had to see that it is a challenge since it is in a few households that we have had these problems "FDG5_F_Abena |
| --- |
